# Contribution of main causes of death to social inequalities in mortality in the whole population of Scania, Sweden

**DOI:** 10.1186/1471-2458-6-79

**Published:** 2006-03-28

**Authors:** Maria Rosvall, Basile Chaix, John Lynch, Martin Lindström, Juan Merlo

**Affiliations:** 1 Department of Health Sciences, Lund University, Malmö University Hospital, Malmö, Sweden; 2 Department of Clinical Sciences, Lund University, Malmö University Hospital, Malmö, Sweden; 3 Research Unit in Epidemiology, Information Systems, and Modelisation (INSERM U707), National Institute of Health and Medical Research, Paris, France; 4 Department of Epidemiology, Biostatistics and Occupational Health, McGill University, Montreal, Canada

## Abstract

**Background:**

To more efficiently reduce social inequalities in mortality, it is important to establish which causes of death contribute the most to socioeconomic mortality differentials. Few studies have investigated which diseases contribute to existing socioeconomic mortality differences in specific age groups and none were in samples of the whole population, where selection bias is minimized. The aim of the present study was to determine which causes of death contribute the most to social inequalities in mortality in each age group in the whole population of Scania, Sweden.

**Methods:**

Data from LOMAS (Longitudinal Multilevel Analysis in Skåne) were used to estimate 12-year follow-up mortality rates across levels of socioeconomic position (SEP) and workforce participation in 975,938 men and women aged 0 to 80 years, during 1991–2002.

**Results:**

The results generally showed increasing absolute mortality differences between those holding manual and non-manual occupations with increasing age, while there were inverted u-shaped associations when using relative inequality measures. Cardiovascular diseases (CVD) contributed to 52% of the male socioeconomic difference in overall mortality, cancer to 18%, external causes to 4% and psychiatric disorders to 3%. The corresponding contributions in women were 55%, 21%, 2% and 3%. Additionally, those outside the workforce (i.e., students, housewives, disability pensioners, and the unemployed) showed a strongly increased risk of future mortality in all age groups compared to those inside the workforce. Even though coronary heart disease (CHD) played a major contributing role to the mortality differences seen, stroke and other types of cardiovascular diseases also made substantial contributions. Furthermore, while the most common types of cancers made substantial contributions to the socioeconomic mortality differences, in some age groups more than half of the differences in cancer mortality could be attributed to rarer cancers.

**Conclusion:**

CHD made a major contribution to the socioeconomic differences in overall mortality. However, there were also important contributions from diseases with less well understood mechanistic links with SEP such as stroke and less-common cancers. Thus, an increased understanding of the mechanisms connecting SEP with more rare causes of disease might be important to be able to more successfully intervene on socioeconomic differences in health.

## Background

It is important to understand the cause-specific structure of social inequalities in mortality for two related reasons. Firstly, it is important to recognize that social inequalities in health are contingent on mechanistic links between social position and the major risk factors for different causes of death [1] and on the social distribution of such risk factors. As such, the strength and patterning of social inequality in mortality will differ across age, causes, and over place and time [2,3]. It follows that, secondly, to more efficiently reduce social inequalities in mortality, it is important to establish which causes of death contribute the most to socioeconomic mortality differentials. Earlier studies have investigated patterns of the main causes of death, i.e., diseases or external causes, to socioeconomic inequalities in mortality [4-14]. In summary, these studies generally showed higher mortality rates among those in lower SEP compared to those in higher SEP, with regard to causes of death such as CVD, cancer, diabetes, chronic obstructive pulmonary disease (COPD) and external causes. However, few studies have specifically investigated which diseases contribute the most to existing socioeconomic mortality differences in specific age groups [9,14] and none were in samples of the whole population, where selection bias is minimized. Since specific causes of death differ by age, the disease entities that contribute most to existing disparities in overall mortality can be expected to differ by age.

In the present study we investigated the contribution of specific causes of death to social inequalities in mortality in the whole population of Scania, Sweden, in ages 0 to 80 years, during 1991 to 2002. The aim of the study was to determine which causes of mortality contribute the most to the differences in overall mortality in each age group. Social inequalities in mortality were measured in two different populations: (1) among those inside the workforce, comparing mortality differences between manual and non-manual occupations, and (2) among the whole population, comparing mortality differences between those inside and outside the workforce. The social situation of those aged 20 years or less was measured according to household occupational status.

## Methods

### Study population

With approval and assistance from Statistics Sweden and the Centre for Epidemiology (Swedish National Board of Health and Welfare) as well as allowance from the Regional Ethical Review Board in Lund, a 43 year (1960–2003) longitudinal database – LOMAS (Longitudinal Multilevel Analysis in Skåne) – has been assembled that includes all the inhabitants in the county of Skåne (about one million). The present study is based on a sub-cohort of the large LOMAS database and included those men (n = 483,943) and women (n = 498,341) who were born between 1910 and 1990 and were alive in January, 1^st ^1991, of whom 99% (n = 975,938) had complete data on SEP and represented the study population.

For every individual we obtain information on causes of death from the National Mortality Register [15] at the Centre for Epidemiology (Swedish National Board of Health and Welfare) [16]. Statistics Sweden [17] provided information on the composition of the household as well as individual occupation from the Swedish National Censuses performed in 1970, 1980, 1985 and 1990. The response rates of these censuses were more than 97 % in each investigation. A unique 10 digit personal identification number, assigned to each person in Sweden for the duration of their lifetime, was used for record linkage between the different registers.

### Socioeconomic position

The data on occupation provided by Statistics Sweden from the census of 1990 yielded information on household occupational status (i.e., the highest occupational level in the household) on subjects who were 20 years old or less and the subject's own occupation among subjects at ages 21–64 years. For those aged 65 years or more (retirement age in Sweden), data on occupation was taken from the last census before retirement, i.e., the 1985 census for those aged 65–69 years, the 1980 census for those aged 70–74 years and the 1970 census for those aged 75–80 years. Job titles and work tasks formed the basis for the classification into standard socioeconomic index (SEI) groups, according to the criteria of Statistics Sweden [18]. SEI classifications take into consideration the educational background needed, additional employment prerequisites, job responsibility levels, and specific duties to be performed. The SEI groups were combined into five categories: non-manual employees (e.g., engineers with university degrees, college teachers, registered nurses, computer operators, office assistants, secretaries), manual workers (e.g., auto mechanics, metal workers, factory workers, check-out assistant, waiters, janitorial staff), self-employed persons (owners of businesses), farmers and those inside the workforce with missing information on occupation (unclassified).

### Workforce participation

The censuses of 1980, 1985 and 1990 provided information on workforce participation, i.e., whether an individual was inside or outside the workforce. Depending on the age of the individual, workforce participation was assessed as either workforce participation of the household (among those aged 20 years or less), own workforce participation (among those aged 21–64 years), or workforce participation before retirement (among those aged 65–70 years). Those categorized as being inside the workforce included the SEI-groups mentioned above, i.e., non-manual employees, manual workers, self-employed persons (owners of businesses), farmers and those inside the workforce with missing information on occupation (unclassified). Those categorized as being outside the workforce included homemakers, the unemployed, students and those receiving a disability pension. Since the census of 1970 used a different categorization of workforce participation, all analyses regarding workforce participation was restricted to those aged 0–70 years.

### Mortality

Information on mortality, was obtained by linking the LOMAS records with the Swedish Causes of Death Register [15]. Each deceased person in Sweden gets one underlying cause of death on the death certificate. An underlying cause of death is defined as either (a) the disease or injury that initiated the chain of diseases that finally resulted in death or (b) the circumstances involving the accident or the act of violence that caused the lethal injury. These underlying causes of death were used in the categorization into major groups of causes of death and specific causes of death. The study population was followed with regard to all-cause mortality, major groups of causes of death (cardiovascular, cancer, external causes, psychiatric mortality and chronic obstructive pulmonary disease (COPD)), and specific causes of death within the major groups of death (coronary heart disease and stroke within CVD mortality; lung cancer, prostate cancer, breast cancer, and colorectal cancer within cancer mortality; and suicides and traffic accidents within mortality due to external causes).

Underlying causes of death were coded in accordance with the 8^th^, 9^th ^and 10^th ^version of the International Classification of Diseases (ICD): Cardiovascular disease (codes 390 to 459 (ICD 8th and 9th version) or I00 to I99 (ICD-10th version)), coronary heart disease (410 to 414 (ICD 8th and 9th version) or I20 to I25 (ICD-10th version)), stroke (430 to 438 (ICD 8th and 9th version) or I60 to I69 (ICD-10th version)), cancer (140 to 239 (ICD 8th and 9th version) or C00 to D48 (ICD-10th version)), lung cancer (162 (ICD 8th and 9th version) or C34 (ICD-10th version)), prostate cancer (185 (ICD 8th and 9th version) or C61 (ICD-10th version)), breast cancer (174 (ICD 8th and 9th version) or C50 (ICD-10th version)), colorectal cancer (153 to 154 (ICD 8th and 9th version) or C18 to C22 (ICD-10th version)), psychiatric diseases (290 to 319 (ICD 8th and 9th version) or F00 to F99 (ICD-10th version)), chronic obstructive pulmonary disease (COPD) (490 to 496 (ICD 8^th ^and 9^th ^version) or J40 to J47 (ICD-10^th ^version)), external causes (800 to 999 (ICD 8th and 9th version) or S00 to T98 and V01 to Y98 (ICD-10th version)), suicide (E 950 to E959 (ICD 8th and 9th version) or X60 to X84 (ICD-10th version)) and traffic accidents (E807 to E846 (ICD 8th and 9th version) or V01 to V99 (ICD-10th version)).

### Statistical methods

Each individual was followed from January 1^st ^1991 until December 31^st ^2002, or death. Relative differences in mortality by SEP/workforce participation were assessed by age-adjusted hazard rate ratios (HRR) presented separately for each age group and sex. We calculated absolute rate differences (ARD) in mortality between manual and non-manual groups/those outside and inside the workforce (expressed as deaths per 100 000 person-years at risk) separately for each age group and sex. The contribution of a specific cause of death to differences in overall mortality by SEP/workforce participation was determined by expressing the rate difference of that specific cause (ARD_S_) as a percentage of the rate difference of total mortality (ARD_T_), i.e., ARD_S_/ARD_T_*100.

## Results

Overall the main causes of death were CVD, cancer, external causes, COPD and psychiatric disorders. With the exception of COPD–which was only present in older age groups, with too few cases at younger ages– these were the causes specifically studied in further analyses. There were 98294 cases of death from all causes (n = 53932 for men and n = 44362 for women). Among these, there were 43678 cases of cardiovascular death (n = 24462 for men and n = 19216 for women), 28895 cases of cancer death (n = 15422 for men and n = 13473 for women), 3295 cases of death due to external causes (n = 2099 for men and n = 1196 for women), and 2816 cases of psychiatric death (n = 1333 for men and n = 1483 for women). The unadjusted rates of CVD, cancer, external causes, psychiatric and all-cause mortality, were 448, 282, 38, 24 and 988 per 100 000 person-years for men and 340, 236, 21, 26 and 770 per 100 000 person-years for women.

### Those inside the workforce

**Figure 1:**
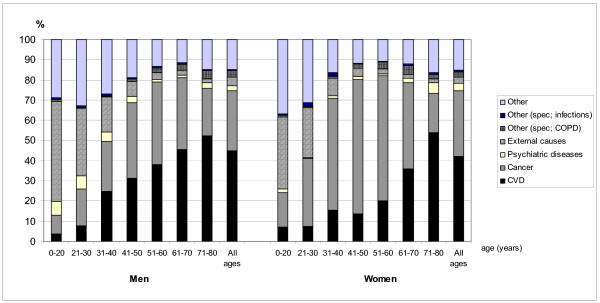
Proportional (%) specific causes of death among men and women inside the workforce holding non-manual or manual occupations in Scania, Sweden. International Classification of Diseases (ICD) codes; cardiovascular disease (codes 390 to 459 (ICD 8^th ^and 9^th ^version) or I00 to I99 (ICD-10^th ^version)), cancer (140 to 239 (ICD 8^th ^and 9^th ^version) or C00 to D48 (ICD-10^th ^version)), psychiatric diseases (290 to 319 (ICD 8^th ^and 9^th ^version) or F00 to F99 (ICD-10^th ^version)), external causes (800 to 999 (ICD 8^th ^and 9^th ^version) or S00 to T98 and V01 to Y98 (ICD-10^th ^version)), infectious diseases (000 to 139 (ICD 8^th ^and 9^th ^version) or A00 to B99 (ICD-10^th ^version)), and COPD (490 to 496 (ICD 8^th ^and 9^th ^version) or J40 to J47 (ICD-10^th ^version)).

The rather small groups of self-employed (n = 37 542; 4.5%), farmers (n = 19 200; 2.3%) and those with missing information on occupation (n = 28 129; 3.4%) were excluded from the analyses due to too few cases in each age-cause group. The specific proportional causes of death in men and women holding manual or non-manual occupations in different age groups are shown in Figure 1. While external causes of death were prominent at younger ages, the most common cause of death in middle-age was cancer and the most common cause of death among the elderly was CVD. The contributions of specific causes of death to overall mortality were somewhat different in men and women. For example, in women, cancer diseases contributed to about 55–65% of all deaths between the ages of 31 to 60 years, while the corresponding percentages in men were 25–40%.

As shown in Table 1, male manual workers had increased hazard rates compared to non-manual employees in all age groups, with HRRs varying from 1.2 (95 % CI:1.1, 1.2) in the oldest age group (71–80 years) up to 1.6 (95 % CI:1.4, 1.9) and 1.6 (95% CI: 1.5, 1.7) among those aged 31–40 years and 41–50 years, respectively. However, the absolute socioeconomic difference in mortality rate increased with increasing age and was greatest in the 71–80 age group, 1780 per 100 000 person-years and smallest in the 0–20 age group, 9.7 per 100 000 person-years. A similar pattern of association was seen in women, however, the relative and absolute differences were less pronounced than in men, and, in general, women showed lower mortality rates than men.

Cause specific mortality rates for major groups of disease and absolute as well as relative mortality differences between those holding non-manual and manual occupations are shown in additional file: 1, table 2. Generally, those holding manual occupations had higher cause-specific mortality rates than those holding non-manual occupations, with the exception of CVD in younger men and cancer, external causes and psychiatric mortality in older women. In the young and middle-aged, the highest relative inequality was seen for psychiatric diseases in both men and women, while at older ages the highest relative inequality was seen for external causes in men and CVD in women. In both men and women of all ages, the absolute inequality between non-manual and manual workers was highest for CVD. Cardiovascular mortality followed a similar pattern to that seen for overall mortality, with the shallowest HRRs and the greatest absolute socioeconomic differences in the older age groups in both men and women. A similar – but weaker – pattern of association was seen for mortality due to cancer, external causes and psychiatric disorders in men, whereas in women, non-manual employees had higher mortality rates than manual workers in older age groups. There was a rather consistent pattern of greater absolute socioeconomic mortality differences in men than in women across the specific causes of death. However, the pattern when using relative inequality measures was less consistent. For example, men and women had rather similar HRRs for mortality due to CVD and psychiatric disorders.

additional file: 2, table 3, shows the contribution of specific causes of death to the socioeconomic differences in all-cause mortality by age group and sex. The contribution of CVD rose with age, whereas the opposite pattern was seen for external causes and psychiatric disorders. The contribution of cancer was the greatest in middle-age and fell with increasing age. There was a larger contribution of CVD at younger and older ages among women than among men, with a negative contribution of cancer in older ages, and a lesser contribution of psychiatric disorders at younger ages. In the 21–30 age group, suicide made up nearly 20 % of the differences in overall mortality between non-manual and manual workers in both men and women. In agreement with the distribution of the main causes of death, CVD and cancer made the largest contributions to differences in total mortality between those holding manual and non-manual occupations. Among men aged 80 years or less, CVD made up 52% of the difference in overall mortality, cancer 18%, external causes 5% and psychiatric disorders 3%. The corresponding contributions in women were 55%, 21%, 2% and 3%, respectively. However, there was no strict correlation between the distribution of main causes of death in various age groups and sexes and the main contributing specific causes of death to the socioeconomic mortality differences. For example, in women, cancer caused around 55–65% of all deaths between the ages 31 and 60 years (Figure 1), but cancer only contributed between 8–40% of the socioeconomic mortality differences in these age groups (table 3). On the other hand, in younger age groups (below age 40) "other causes of death", i.e., non-major causes of death, caused about 15–40 % of all deaths, while "other causes of death" contributed to between 32 and 61% of the mortality differences by SEP.

### Whole population

**Figure 2:**
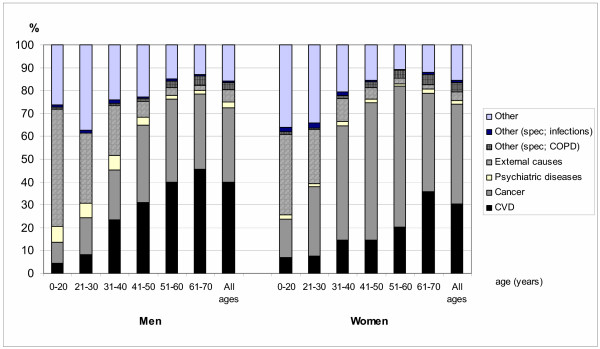
Proportional (%) specific causes of death in the whole population of men and women in Scania, Sweden. International Classification of Diseases (ICD) codes; cardiovascular disease (codes 390 to 459 (ICD 8^th ^and 9^th ^version) or I00 to I99 (ICD-10^th ^version)), cancer (140 to 239 (ICD 8^th ^and 9^th ^version) or C00 to D48 (ICD-10^th ^version)), psychiatric diseases (290 to 319 (ICD 8^th ^and 9^th ^version) or F00 to F99 (ICD-10^th ^version)), external causes (800 to 999 (ICD 8^th ^and 9^th ^version) or S00 to T98 and V01 to Y98 (ICD-10^th ^version)), infectious diseases (000 to 139 (ICD 8^th ^and 9^th ^version) or A00 to B99 (ICD-10^th ^version)), and COPD (490 to 496 (ICD 8^th ^and 9^th ^version) or J40 to J47 (ICD-10^th ^version)).

The specific proportional causes of death in the whole population stratified by age group were very similar to the ones seen for those inside the workforce holding manual or non-manual occupations (Figure 2).

Table 4 presents overall mortality rates as well as absolute and relative mortality differences by workforce participation, i.e., between those inside and outside the workforce, in different age groups. Those outside the workforce showed markedly higher mortality rates compared to those inside the workforce at all ages in both men and women, with the highest HRRs being 3.1 (95 % CI: 2.7, 3.5) at ages 31–40 years and 2.4 (95 % CI:2.2, 2.6) at ages 41–50 years, and the lowest being 1.5 (95 % CI:1.5, 1.6) for men and 1.4 (95% CI: 1.3, 1.4) for women at ages 61–70 years. The absolute inequalities were generally bigger than those seen between subjects holding manual or non-manual occupations, and increased with increasing age, with the exception of the oldest age group (61–70 years). Generally, women showed lower mortality rates than men. Furthermore, the relative and absolute differences in mortality were less pronounced in women than in men.

Additional file: 3, table 5, presents cause-specific mortality rates for major groups of disease as well as absolute and relative mortality differences by workforce participation, i.e., between those inside and outside the workforce, respectively, in different age groups. Those outside the workforce generally showed higher cause-specific mortality rates than those inside the workforce, with the highest HRRs seen for psychiatric disorders, and the greatest absolute differences seen for CVD and cancer. Generally, the relative differences followed an inverted u- or j-shaped association with increasing age, while the absolute differences increased with increasing age until the age of 60. With the exception for cancer mortality between the ages 31 and 50 years, women generally had lower specific mortality rates than men; among those outside the workforce, the male/female ratios were as highest 3.6 for cardiovascular mortality, 1.7 for cancer mortality, 3.5 for mortality due to external causes, and 8.6 for mortality due to psychiatric disorders (data not shown). Similar figures were seen among those inside the workforce. While there was a consistent pattern of bigger absolute differences in mortality in men than in women across the different causes of death, this could only be seen with regard to cancer mortality when using a relative measure of mortality differences.

Additional file: 4, table 6, shows the contribution of specific causes to differences in all-cause mortality by workforce participation, i.e., between those categorized as being inside and outside the workforce, respectively, in different age groups. Among men aged 70 years or less, CVD contributed to 45% of the difference in all-cause mortality between those inside or outside the workforce, cancer to 25%, external causes to 3% and psychiatric disorders to 3%. The corresponding contributions in women were 39%, 31%, 2% and 2%, respectively. CVD and cancer made the largest specific contributions to differences in overall mortality, however, the relative contributions were less pronounced than those seen in the analyses of individuals with manual or non-manual occupations. In men, the contributions of CVD and cancer rose with age, while the contributions of external causes and psychiatric disorders fell. A similar pattern was seen in women, with the exception of cancer, which made a constant contribution of around 15–20% in all ages between 21 and 70 years. The consistent negative contribution of breast cancer across the age groups seen for mortality differences between manual and non-manual female workers could not be seen for mortality differences with regard to workforce participation. Furthermore, the negative contribution of cancer among older women was not present. The extent to which the absolute differences in mortality could be explained by the mentioned specific causes of death was also less pronounced in each age group, with a larger proportion being explained by other causes of death. For example in the 61–70 age group, socioeconomic differences in cardiovascular mortality could explain about 85 % of the female difference in all-cause mortality seen between those holding manual or non-manual occupations, but only about 50% of the female difference in all-cause mortality between those inside or outside the workforce. The contributing role of external causes to the mortality differences at younger ages was also less pronounced than with regard to the differences seen between those holding manual or non-manual occupations.

**Figure 3:**
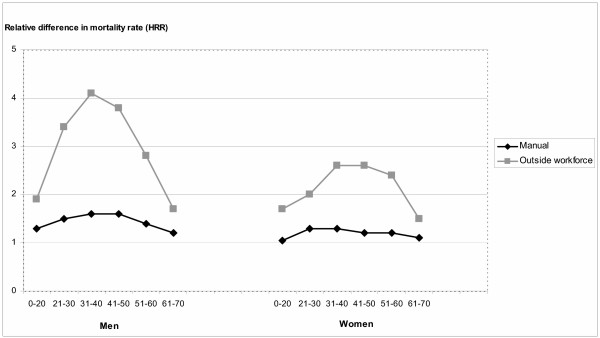
Age-adjusted relative difference measured as hazard rate ratios (HRR) in all-cause mortality between those holding non-manual occupations, those holding manual occupations and those outside the workforce, in Scania, Sweden. Those holding non-manual occupations were used as the reference. Variations by age and sex are presented.

**Figure 4:**
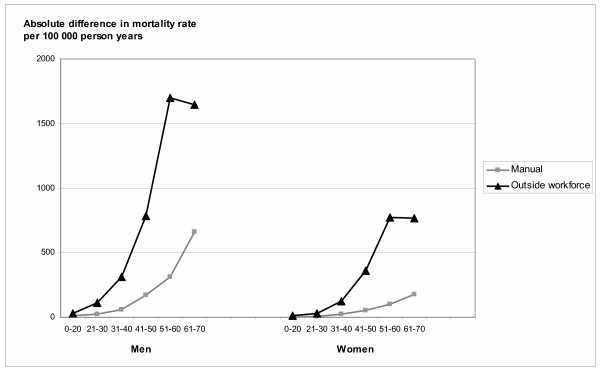
Absolute difference in all-cause mortality rate per 100 000 person-years between those holding non-manual occupations, those holding manual occupations and those outside the workforce, in Scania, Sweden. Those holding non-manual occupations were used as the reference group. Variations by age and sex are presented.

We also conducted analyses comparing the overall mortality of those outside the workforce to those with non-manual occupations. As seen in Figure 3, the relative mortality differences between those outside the workforce and those with non-manual occupations were greater than the differences seen between those with manual or non-manual occupations in all age groups in both men and women. A similar pattern was seen for the absolute differences in overall mortality, as shown in Figure 4.

## Discussion

In our study, those holding manual occupations generally had higher cause-specific mortality rates than those holding non-manual occupations. With the exception of younger age groups (40 years or less), CVD and cancer made the largest contributions to these mortality differences. As expected, absolute socioeconomic differences increased with age, while there were inverted u-shaped associations between SEP and mortality when using relative inequality measures. The decline of relative inequality with age is partly a result of the increasing absolute death rates in both socioeconomic groups. Furthermore, while absolute mortality differences were consistently greater in men than in women across the specific causes of death, the relative inequalities in mortality were rather similar for CVD and psychiatric disorders. Thus, the results may point in different directions depending on the choice of measure of effect (i.e., relative vs. absolute), leaving room for misinterpretations. One solution, as illustrated by our study, is to present absolute figures (e.g., rates) besides relative or absolute differences [19,20]. It has been argued that from the perspective of population health prevention it is more useful to focus on absolute levels and differences, while relative measures are more appropriate for etiological investigations [21]. However, even if etiology can be established, attention should inevitably turn to the question of "how big" or "how important" a particular cause may be. Thus, it is still useful to look at absolute measures, since conclusions about the importance of various risk factors can be different depending on which scale is used to measure social inequalities [1].

In agreement with the distribution of main causes of death, CVD and cancer made the largest contributions to differences in overall mortality between those holding manual and non-manual occupations. The relative inequalities found in our study correspond well to those found in the study by Kunst et al. of socioeconomic mortality differences in men aged 45–59 years [9]. In that study, the relative differences between Swedish non-manual and manual workers with regard to overall mortality, CVD, cancer and external causes were 1.41, 1.36, 1.18 and 1.76, respectively. The corresponding relative inequalities among men aged 51–60 years in our study were 1.4, 1.4, 1.3 and 1.7. The contribution of specific causes of death to excess mortality by SEP changed substantially with age, with CVD becoming more important at older ages, whereas the opposite pattern was seen for psychiatric disorders and external causes. This is probably mostly due to the differing distributions of main causes of death at different ages. The contribution of cancer was greatest in middle-age, and fell with increasing age. It has been argued that the diminished contribution of cancer among older age groups is partly related to the distribution patterns of lungcancer and breastcancer. Subjects in older age groups with higher SEP have lungcancer to a similar extent to subjects with lower SEP, due to the reversal in the socioeconomic pattern of smoking, which took place from the 1940s through the 1960s in the Western world [14,22]. Additionally, earlier studies have shown that the most common type of cancer seen in women, namely breast cancer, is more common in higher SEP groups [13,14,23], even though some studies have shown that socioeconomic differences in breast cancer mortality are currently changing and the previously observed positive gradient has disappeared [24]. However, we found no strict correlation between the distribution of main causes of death in various age groups and sexes and the main contributing specific causes of death to the socioeconomic mortality differences. For example, even though CVD and cancer made similar contributions to overall mortality in older men and women, the contribution of CVD to the socioeconomic mortality differences was greater in women than in men, while the contribution of cancer was smaller. Similar results were seen in a large collaborative study including countries in southern and northern Europe [14].

The results regarding the proportional role of specific causes of death are of importance for understanding SEP inequalities in overall mortality in various age groups and sex-specific groups, since they might broaden ideas about potential preventive strategies for reducing social inequalities in health. Our study shows that different causes of death with widely different mechanistic links with SEP have different influence on social inequalities in health in different age groups. Thus, the focus of preventive programs to reduce social inequalities in health should vary by age. Our study lends support to the well-known association between SEP and CHD, with CHD playing a major contributing role to the mortality differences seen. This might be partly due to the fact that cardiovascular diseases constitute about half of all deaths in Sweden, with CHD being the largest disease entity within the category of cardiovascular diseases. Furthermore, CHD has well established mechanistic links with SEP, through smoking, dyslipidemia, and hypertension. However, as in the international study by Huisman et al. [14], stroke and other types of cardiovascular diseases also made substantial contributions to the mortality differences. For these kinds of diseases the mechanistic links with SEP are somewhat less well-established [25,26]. Additionally, while the most common types of cancers – lung, colorectal, prostate and breast cancer – made substantial contributions to the socioeconomic mortality differences, in some age groups more than half of the differences in cancer mortality could be attributed to rarer cancers. Less-common diseases also seemed to play a major role in explaining the socioeconomic mortality gradient in younger ages. For example, 27% of the male and 40% of the female manual/non-manual inequalities in deaths before age 50 were due to "other causes". Thus, an increased understanding of the mechanisms connecting SEP with diseases that are less common than CHD might also be important as a basis for more successful intervention regarding socioeconomic health differences.

Being outside the workforce was associated with a strongly increased risk of future mortality compared to being inside in all age groups in both men and women. When interpreting this increased risk it is important to remember that the former group was heterogeneous and included, for example, students, who would be expected to have relatively low mortality rates, as well as housewives and disability pensioners. These mortality differences were generally greater than the differences seen between those holding manual or non-manual occupations. When comparing mortality rates between those outside the workforce and those with non-manual occupations, the differences were even bigger. This was especially true when using absolute measures of effect, but it was also the case when using relative inequality measures. Thus, the inequalities in mortality seem to be greater according to workforce participation than according to occupational category. The contributions of specific causes of death to differences in total mortality between those inside and outside the workforce were different from those seen when comparing manual and non-manual occupations. Even though CVD made a pronounced contribution to the mortality differences, the roles of other specific causes besides cardiovascular, cancer, external causes, or psychiatric diseases were relatively greater according to workforce participation than according to occupational category. These other causes mainly included endocrinological and alcohol-related diseases in younger people and the middle-aged, and COPD and infections among the elderly (data not shown). Furthermore, with the exception of younger ages, breast cancer showed higher mortality rates among women outside the workforce than in women inside the workforce.

The term socioeconomic position has been argued to encompass the social and economic factors that influence what positions individuals hold within a society [27]. The most well-developed conceptual framework in social epidemiology is the Weberian approach to social stratification, where the key linkage with health is the distribution of skills, knowledge and resources [27-29]. The stratification scheme used in our study, as a measure of SEP, was elaborated by Statistics Sweden and has been used as a standard for national demographic statistics for several decades. The classification is in agreement with the Erikson, Goldthorpe and Portocarero (EGP) scheme used in the study by Kunst et al[9] based on work tasks, job responsibility levels and employment relations, but also on the educational background needed [18]. The cross-sectional measurement of occupational status and relation to the workforce at one point in time gives room for potential misclassification. People may for example change occupations or move into the workforce after having completed high school or university studies. However, potential misclassification of SEP would be expected to be nondifferential and would thus lead to an underestimate of an effect on mortality. Using a more detailed measure of SEP showed somewhat more pronounced absolute as well as relative socioeconomic differences in overall mortality in each age group in both men and women, however, with similar patterns of associations as seen when using broader categories (data not shown). Additionally, instead of omitting those outside the workforce– constituting a large part of the population in some age groups –from the analyses, or categorizing them according to their latest or longest held occupation, which is often done in epidemiological studies, we have chosen to specifically study the influence on mortality of being outside the workforce, using the same etiological model as used for those holding manual or non-manual occupations. The rational behind this choice is the fact that those outside the workforce have a similar SEP, and generally have more unfavorable life-styles, and social networks, and an increased risk of future chronic disease compared to those inside the workforce [30-33].

Another methodological issue that needs to be addressed is the classification of end-point. Vital status at the end of the follow-up was updated on all individuals by data linkage with Swedish Causes of Death Register, which has an almost complete coverage even for residents who die outside Sweden [15]. Swedish statistics on cause of death are among the longest established worldwide, dating back to 1749 when a nationwide report system was first introduced [15]. The mortality register encompass 97% of all deaths in Sweden, while census participation rates range between 97% and 99%. Thus, there is no reason to believe that incomplete retrieval of cases biased the results. The quality of the data on cause of death also depends on the accuracy of the physician's completion of the death certificate. It is widely known that the data on cause of death are generally more accurate in younger than in older people, since older people often have multiple diseases and so it can be harder to determine the underlying cause of death. Younger people are also more frequently autopsied after death than older people [15]. Such misclassification might theoretically have an effect on the absolute levels for different causes of death, and where the use of more narrow classification categories such as specific causes of death would be more prone to misclassification errors than that using broader categories such as major causes of death. However, there is no obvious reason to believe that this kind of potential misclassification would differ by SEP in a country like Sweden with a well-developed social security system. One of the strengths of our study is that our data covers the total general population in the county of Skåne, minimizing the risk of selection bias. Thus, the problem with only the healthiest people attending the study potentially attenuating the associations studied that is often seen with general population based studies using population samples, is not present here [34].

## Conclusion

In conclusion, the results showed that individuals holding manual occupations generally had higher cause-specific mortality rates than those holding non-manual occupations. In agreement with the distribution of main causes of death, CVD and cancer made the largest contributions to differences in overall mortality between those holding manual and non-manual occupations. Additionally, people outside the workforce (i.e., students, housewives, disability pensioners, and the unemployed) showed a strongly increased risk of future mortality in all age groups compared to those inside the workforce. These mortality differences were generally greater than the differences seen between those holding manual and non-manual occupations. The results regarding which specific causes contribute the most to inequalities in overall mortality by SEP in various age groups and sex are of importance since they might offer clues for potential preventive strategies aimed at reducing social inequalities in health. As expected, diseases with well-established mechanistic links with SEP, such as CHD (smoking, dyslipidemia, and hypertension) and lung cancer (smoking) made major contributions to the socioeconomic mortality differences seen. However, there were also important contributions from diseases with less well understood mechanistic links with SEP, such as stroke and other types of CVD and less-common cancers. Moreover, less-common diseases seemed to play a major role in explaining the socioeconomic mortality gradient in younger age groups. Thus, an increased understanding of the mechanisms connecting SEP with more rare causes of death might be important to be able to more successfully intervene on socioeconomic differences in health.

## Competing interests

The author(s) declare that they have no competing interests.

## Authors' contributions

MR, BC and JM are responsible for the conception, design, analysis, and interpretation of the data; the drafting, writing, and revision of the content; and the approval of the final version. JL and ML have each made substantial contributions to the design and interpretation of the data, the revision of the content, and the approval of the final version.

## Pre-publication history

The pre-publication history for this paper can be accessed here:



## Supplementary Material

Additional file 1 Click here for file

Additional file 2 Click here for file

Additional file 3 Click here for file

Additional file 4 Click here for file

## Figures and Tables

**Table 1:** All-cause mortality per 100 000 person-years by socioeconomic group among those inside the workforce stratified by age groups. A total population investigation from Scania, Sweden.

Age group (years)	n (%)	Socioeconomic group*	Deaths (per 100 000 person-years)	Relative difference Hazard rate ratio (95 % CI)	Absolute difference
***Men***					
0–20	72263 (56)	Non-manual	34		
	48158 (38)	Manual	43.3	1.3 (1.1, 1.5)	9.7
21–30	17130 (29)	Non-manual	52		
	34646 (59)	Manual	74.6	1.5 (1.2, 1.9)	22.6
31–40	25934 (43)	Non-manual	105		
	27311 (45)	Manual	163	1.6 (1.4, 1.9)	58
41–50	32026 (47)	Non-manual	277		
	26748 (39)	Manual	447	1.6 (1.5, 1.7)	170
51–60	19973 (44)	Non-manual	859		
	18499 (41)	Manual	1174	1.4 (1.3, 1.5)	315
61–70	13525 (41)	Non-manual	2607		
	12503 (39)	Manual	3266	1.2 (1.2, 1.3)	659
71–80	9572 (27)	Non-manual	7563		
	13374 (38)	Manual	9343	1.2 (1.1, 1.2)	1780
					
***Women***					
0–20	69578 (57)	Non-manual	18		
	45930 (38)	Manual	20.2	1.0 (0.9, 1.4)	2.2
21–30	22699 (40)	Non-manual	33.1		
	27788 (49)	Manual	39.7	1.3 (1.0, 1.7)	6.6
31–40	30411 (52)	Non-manual	85		
	23853 (40)	Manual	108	1.3 (1.1, 1.6)	23
41–50	34840 (53)	Non-manual	219		
	25541 (39)	Manual	275	1.2 (1.1, 1.3)	56
51–60	18941 (45)	Non-manual	489		
	19510 (46)	Manual	587	1.2 (1.1, 1.3)	98
61–70	9960 (38)	Non-manual	1407		
	12529 (48)	Manual	1586	1.1 (1.02, 1.2)	179
71–80	10800 (33)	Non-manual	5525		
	14336 (48)	Manual	6112	1.2 (1.1, 1.2)	587

* Those classified as being inside the workforce holding non-manual or manual occupations; non-manual employees (e.g., engineers with university degrees, college teachers, registered nurses, computer operators, office assistants, salespeople, secretaries), manual workers (e.g., auto mechanics, metal workers, factory workers, check-out assistant, waiters, janitorial staff). Those aged 20 years or less were classified according to household occupational status.

**Table 4:** All-cause mortality per 100 000 person-years by workforce participation stratified by age groups in Swedish men and women. A total population investigation from Scania, Sweden.

Age group	n (%)	Workforce participation†	Deaths (per 100 000 person-years)	Relative difference HRR (95 % CI)	Absolute difference
***Men***					
0–20	127313 (95.5)	Inside workforce	38		
	6046 (4.5)	Outside workforce	61	1.7 (1.2, 2.3)	23
21–30	58340 (84.7)	Inside workforce	68		
	10541 (15.3)	Outside workforce	162	2.5 (2.1, 3.0)	94
31–40	60638 (91.1)	Inside workforce	138		
	5958 (8.9)	Outside workforce	415	3.1 (2.7, 3.5)	277
41–50	68088 (92.1)	Inside workforce	362		
	5835 (7.9)	Outside workforce	1061	2.9 (2.7, 3.2)	699
51–60	45372 (86.1)	Inside workforce	1018		
	7330 (13.9)	Outside workforce	2559	2.4 (2.2, 2.5)	1541
61–70	33178 (65.6)	Inside workforce	2917		
	17363 (34.4)	Outside workforce	4255	1.5 (1.5, 1.6)	1338
					
***Women***					
0–20	122030 (95.3)	Inside workforce	19		
	5988 (4.7)	Outside workforce	31	1.7 (1.1, 2.6)	12
21–30	56222 (82.7)	Inside workforce	37		
	11733 (17.3)	Outside workforce	60	1.7 (1.3, 2.2)	23
31–40	58973 (87.8)	Inside workforce	96		
	8177 (12.2)	Outside workforce	209	2.3 (1.9, 2.7)	113
41–50	65700 (89.4)	Inside workforce	241		
	7750 (10.6)	Outside workforce	581	2.4 (2.2, 2.6)	340
51–60	42447 (78.1)	Inside workforce	541		
	11917 (21.9)	Outside workforce	1261	2.2 (2.0, 2.3)	720
61–70	26260 (45.8)	Inside workforce	1508		
	31096 (54.2)	Outside workforce	2173	1.4 (1.3, 1.4)	665

*HRR, hazard rate ratio; CI, confidence interval.

† Those inside the workforce include: non-manual employees (e.g., engineers with university degrees, college teachers, registered nurses, computer operators, office assistants, salespeople, secretaries), manual workers (e.g., auto mechanics, metal workers, factory workers, check-out assistant, waiters, janitorial staff), self-employed persons (owners of businesses), farmers and those inside the workforce with missing information on occupation (unclassified). Those who were categorized as being outside the workforce include homemakers, unemployed, students and those who had a disability pension. Those aged 20 years or less were classified according to the relation to the workforce of the household.
