# Supplementary material for: Contribution of main causes of death to social inequalities in mortality in the whole population of Scania, Sweden
Source: BMC Public Health. 2006 Mar 28;6:79. doi: 10.1186/1471-2458-6-79 (PMC1513205; doi:10.1186/1471-2458-6-79)
Supplement: Supplementary file 1 [file 1471-2458-6-79-s1.doc]

TABLE 2. Cause specific mortality for major groups of diseases per 100 000 person-years by socioeconomic group stratified by age groups in Swedish men and women inside the workforce in a total population investigation from Scania, Sweden.

		**Cardiovascular mortality†**			**Cancer mortality†**			**External causes mortality†**			**Psychiatric mortality†**		
Age group (years)	Socioeconomic group‡	Deaths (per 100 000 person-years)	Relative difference HRR (95 % CI)*	Absolute difference	Deaths (per 100 000 person-years)	Relative difference HRR (95 % CI)*	Absolute difference	Deaths (per 100 000 person-years)	Relative difference HRR (95 % CI)*	Absolute difference	Deaths (per 100 000 person-years)	Relative difference HRR (95 % CI)*	Absolute difference
Men													
0-20	Non-manual	2.1			3.2			17			2		
	Manual	1.8	0.8 (0.4, 1.8)	-0.3	4.0	1.2 (0.7, 2.1)	0.8	22	1.3 (1.01, 1.6)	5	3.5	1.7 (0.9, 3.3)	1.5
21-30	Non-manual	6.8			11.7			15.6			3		
	Manual	4.3	0.7 (0.3, 1.4)	-2.5	12.6	1.1 (0.7, 1.9)	0.9	25.8	1.6 (1.1, 2.4)	10.2	5	1.8 (0.7, 4.5)	2
31-40	Non-manual	24			31			20			3		
	Manual	42	1.8 (1.4, 2.4)	18	35	1.2 (0.9, 1.6)	4	28	1.4 (1.03, 2.0)	8	10	3.7 (1.7, 8.1)	7
41-50	Non-manual	76			115			22			6		
	Manual	153	2.0 (1.7, 2.3)	77	154	1.3 (1.2, 1.5)	39	33	1.5 (1.1, 2.0)	11	15	2.5 (1.5, 4.1)	9
51-60	Non-manual	324			361			27			8		
	Manual	452	1.4 (1.3, 1.6)	128	468	1.3 (1.2, 1.4)	107	45	1.7 (1.2, 2.3)	18	19	2.3 (1.3, 3.9)	11
61-70	Non-manual	1124			1018			41			43		
	Manual	1550	1.4 (1.3, 1.4)	426	1068	1.0 (1.0, 1.1)	50	69	1.7 (1.2, 2.3)	28	45	1.1 (0.7, 1.5)	2
71-80	Non-manual	3895			1872			144			225		
	Manual	4937	1.2 (1.2, 1.3)	1042	2131	1.1 (1.04, 1.2)	259	187	1.3 (1.01, 1.6)	43	245	1.1 (0.9, 1.3)	20
Women													
0-20§	Non-manual	1.1			3			6.5			0.2		
	Manual	1.6	1.6 (0.7, 4.1)	0.5	3.1	1.0 (0.5, 1.8)	0.1	7.1	1.1 (0.7, 1.7)	0.6	0.5	2.2 (0.4, 13.2)	0.3
21-30	Non-manual	1.8			12.5			8.5			—		
	Manual	3.3	1.9 (0.7, 5.4)	1.5	12.2	1.0 (0.7, 1.7)	-0.3	9.6	1.2 (0.7, 2.0)	1.1	0.3	—	0
31-40	Non-manual	12.2			52			7.2			0.3		
	Manual	17.2	1.5 (1.0, 2.1)	5.0	53.8	1.1 (0.9, 1.3)	1.8	9.8	1.4 (0.8, 2.3)	2.6	1.8	6.3 (0.7, 54.3)	1.5
41-50	Non-manual	26			154			10			2		
	Manual	43	1.6 (1.3, 2.1)	17	172	1.1 (1.0, 1.2)	18	10.9	1.1 (0.7, 1.7)	0.9	6	2.5 (1.1, 5.6)	4
51-60	Non-manual	91			314			17			4		
	Manual	126	1.4 (1.1, 1.6)	35	353	1.1 (1.01, 1.2)	39	10	0.6 (0.3, 0.97)	-7	5	1.0 (0.4, 2.2)	1
61-70	Non-manual	455			649			32			26		
	Manual	607	1.3 (1.1, 1.4)	152	643	1.0 (0.9, 1.1)	-6	26	0.8 (0.5, 1.3)	-6	37	1.4 (0.9, 2.1)	11
71-80	Non-manual	2868			1144			112			310		
	Manual	3377	1.2 (1.2, 1.3)	509	1137	1.0 (0.9, 1.1)	-7	124	1.2 (0.9, 1.5)	12	289	1.0 (0.9, 1.2)	-21

*HRR, hazard rate ratio; CI, confidence interval.

†International Classification of Diseases (ICD) codes; cardiovascular disease (codes 390 to 459 (ICD 8th and 9th version) or I00 to I99 (ICD-10th version)), cancer (140 to 239 (ICD 8th and 9th version) or C00 to D48 (ICD-10th version)), psychiatric diseases (290 to 319 (ICD 8th and 9th version) or F00 to F99 (ICD-10th version)), and external causes (800 to 999 (ICD 8th and 9th version) or S00 to T98 and V01 to Y88 (ICD-10th version)).

‡ Those classified as being inside the workforce holding non-manual or manual occupations; Non-manual employees (e.g., engineers with university degrees, college teachers, registered nurses, computer operators, office assistants, secretaries), manual workers (e.g., auto mechanics, metal workers, factory workers, check-out assistant,waiters, janitorial staff). Those aged 20 years or less were classified according to household occupational status.
