# Supplementary material for: Contribution of main causes of death to social inequalities in mortality in the whole population of Scania, Sweden
Source: BMC Public Health. 2006 Mar 28;6:79. doi: 10.1186/1471-2458-6-79 (PMC1513205; doi:10.1186/1471-2458-6-79)
Supplement: Supplementary file 2 [file 1471-2458-6-79-s2.doc]

TABLE 3. Contribution (%) of specific causes of death to socioeconomic differences in all-cause mortality in different age-groups among those inside the workforce holding manual or non-manual occupations. A total population from Scania, Sweden.*

Age group (years)	**CVD overall†**	Coronary heart disease‡	Stroke‡	Other CVD	**Cancer overall‡**	Lung-cancer‡	Prostate-cancer‡	Breast-cancer‡	Colorectal-cancer‡	Other types of cancer	**External causes‡**	Suicide	Traffic accidents	Other types of external causes	**Psychiatric diseases‡**	**Other diseases or causes**
Men																
0-20	**-3.2**	2.1	-5.0	-0.3	**8.6**	2.1	0	—	-1.7	8.2	**47.5**	3.2	14.0	30.3	**15.0**	**32.1**
21-30	**-11.1**	-3.1	-2.2	-5.8	**4.0**	2.2	-2.2	—	-3.0	7.0	**45.1**	19.9	1.0	24.2	**8.7**	**53.0**
31-40	**31.0**	17.9	3.4	9.7	**7.0**	1.7	1.0	—	-1.5	5.8	**13.8**	1.9	4.3	7.6	**12.1**	**36.1**
41-50	**45.3**	32.3	3.6	9.4	**22.9**	12.8	0.5	—	-1.8	11.4	**6.5**	2.0	1.0	3.5	**5.3**	**20.0**
51-60	**40.6**	33.0	2.6	5.0	**33.9**	18.5	3.3	—	3.2	8.9	**5.7**	1.3	2.2	2.2	**3.5**	**16.3**
61-70	**64.6**	42.2	9.4	13.0	**7.6**	7.4	0.3	—	-1.4	1.3	**4.2**	0.6	1.5	2.1	**0.3**	**23.3**
71-80	**58.5**	40.0	9.0	9.5	**14.6**	5.6	-0.7	—	2.3	7.4	**2.4**	0.3	0.2	1.9	**1.1**	**23.4**
All ages ≤ 50	**33.8**	23.1	2.3	8.4	**15.8**	8.1	0.4	—	-1.7	9.0	**14.8**	4.5	2.4	7.9	**8.5**	**27.1**
All ages ≤ 70	**47.4**	33.3	4.7	9.4	**16.7**	10.8	0.9	—	-0.5	5.5	**8.8**	2.1	2.2	4.5	**4.4**	**22.7**
All ages ≤ 80	**52.0**	33.6	7.9	10.5	**18.2**	6.9	2.1	—	1.4	7.8	**4.8**	1.0	1.0	2.8	**3.1**	**21.9**
Women																
0-20	**§**	§	§	§	**§**	§	§	§	§	§	**§**	§	§	§	**§**	**§**
21-30	**22.7**	-1.5	12.9	11.3	**-4.5**	-4.2	—	-2.9	-1.4	4.0	**16.7**	19.6	-18.2	15.3	**4.3**	**60.8**
31-40	**21.7**	18.1	2.2	1.4	**7.8**	7.4	—	-1.3	3.9	-2.2	**11.3**	8.6	-1.7	4.4	**6.5**	**52.7**
41-50	**30.3**	24.6	3.9	1.8	**32.1**	28.1	—	-7.1	2.7	8.4	**1.6**	-2.1	3.5	0.2	**7.1**	**28.6**
51-60	**35.7**	26.5	2.0	7.2	**39.8**	9.8	—	-5.1	4.9	30.2	**-7.1**	-0.1	-1.3	-5.7	**1.0**	**30.6**
61-70	**84.4**	68.7	14.8	0.9	**-3.3**	-1.6	—	-1.1	2.2	-2.8	**-3.3**	3.0	-4.1	-2.2	**6.1**	**16.1**
71-80	**86.7**	52.8	12.3	21.6	**-1.2**	-1.0	—	-1.0	-3.4	4.2	**2.0**	0.5	0.5	1.0	**-3.5**	**16.0**
All ages ≤ 50	**28.9**	22.9	2.9	3.1	**16.6**	15.3	—	-6.5	1.8	6.0	**7.6**	4.7	0.6	2.3	**7.1**	**39.8**
All ages ≤ 70	**40.4**	26.8	7.4	6.2	**34.1**	8.5	—	-1.0	4.9	21.7	**1.2**	1.3	-0.4	0.3	**3.6**	**20.7**
All ages ≤ 80	**55.0**	29.9	11.4	13.7	**20.9**	3.9	—	-1.0	3.0	15.0	**1.8**	0.5	0.2	1.1	**3.4**	**18.9**

*The contribution of a specific cause of death to differences in overall mortality by SEP was determined by expressing the rate difference of that specific cause (ARD_S) as a percentage of the rate difference of total mortality (ARD_T), (that is, ARD_S/ARD_T*100).

† CVD, cardiovascular disease

‡ International Classification of Diseases (ICD) codes; cardiovascular disease (codes 390 to 459 (ICD 8th and 9th version) or I00 to I99 (ICD-10th version)), coronary heart disease (410 to 414 (ICD 8th and 9th version) or I20 to I25 (ICD-10th version)), stroke (430 to 438 (ICD 8th and 9th version) or I60 to I69 (ICD-10th version)), cancer (140 to 239 (ICD 8th and 9th version) or C00 to D48 (ICD-10th version)), lung cancer (162 (ICD 8th and 9th version) or C34 (ICD-10th version)), prostate cancer (185 (ICD 8th and 9th version) or C61 (ICD-10th version)), breast cancer (174 (ICD 8th and 9th version) or C50 (ICD-10th version)), colorectal cancer (153 to 154 (ICD 8th and 9th version) or C18 to C22 (ICD-10th version)), psychiatric diseases (290 to 319 (ICD 8th and 9th version) or F00 to F99 (ICD-10th version)), external causes (800 to 999 (ICD 8th and 9th version) or S00 to T98 and V01 to Y98 (ICD-10th version)). suicide (E 950 to E959 (ICD 8th and 9th version) or X60 to X84 (ICD-10th version)) and traffic accidents (E807 to E846 (ICD 8th and 9th version) or V01 to V99 (ICD-10th version)). Those aged 20 years or less were classified according to household occupational status.

§ Too few cases (overall mortality difference=2.2) to be able to look at specific mortality.
