# Supplementary material for: Contribution of main causes of death to social inequalities in mortality in the whole population of Scania, Sweden
Source: BMC Public Health. 2006 Mar 28;6:79. doi: 10.1186/1471-2458-6-79 (PMC1513205; doi:10.1186/1471-2458-6-79)
Supplement: Supplementary file 4 [file 1471-2458-6-79-s4.doc]

TABLE 6. Contribution (%) of specific causes of death to differences in all-cause mortality in different age-groups by workforce participation. A total population from Scania, Sweden.*

Age group (years)	**CVD overall†‡**	Coronary heart disease‡	Stroke‡	Other CVD	**Cancer overall‡**	Lung-cancer‡	Prostate-cancer‡	Breast-cancer‡	Colorectal-cancer‡	Other types of cancer	**External causes‡**	Suicide‡	Traffic accidents‡	Other tyoes of external causes	**Psychiatric diseases‡**	**Other diseases or causes**
Men																
0-20	**-1.7**	-0.9	-1.2	0.4	**8.7**	0	0	—	4.8	3.9	**47.8**	3.0	25.9	18.9	**13.0**	**32.2**
21-30	**9.6**	3.4	1.1	5.4	**5.3**	0.5	-0.1	—	-0.3	5.2	**17.0**	-1.3	0.6	17.7	**13.8**	**54.3**
31-40	**16.2**	11.6	1.3	3.3	**5.4**	1.9	-0.2	—	0.9	2.8	**12.6**	1.9	4.3	6.4	**2.6**	**63.2**
41-50	**33.0**	19.2	4.9	8.9	**13.2**	2.2	-0.1	—	1.9	9.2	**8.4**	2.2	1.4	4.8	**6.9**	**38.5**
51-60	**48.9**	32.0	7.0	9.9	**14.9**	5.1	0	—	1.8	8.0	**3.4**	0.6	0.6	2.2	**3.0**	**29.8**
61-70	**47.9**	34.0	5.4	8.5	**16.9**	8.9	0.3	—	-1.4	9.1	**2.8**	0.5	1.2	1.1	**1.9**	**30.4**
All ages ≤ 50	**24.2**	14.3	2.8	7.1	**9.9**	1.3	0.4	—	1.1	7.1	**11.8**	2.7	1.7	7.4	**9.8**	**44.3**
All ages ≤ 70	**44.7**	29.0	6.6	9.1	**24.6**	6.6	3.0	—	3.0	12.0	**3.1**	0.6	0.8	1.7	**2.7**	**24.9**
Women																
0-20	**0.8**	-2.2	8.2	5.2	**-3.3**	-0.8	—	0	-1.6	-0.9	**26.7**	0.8	6.3	19.6	**9.2**	**66.6**
21-30	**6.0**	3.9	2.2	-0.1	**13.0**	5.2	—	-2.6	4.3	6.1	**5.6**	2.6	4.2	-1.2	**7.8**	**67.6**
31-40	**11.5**	6.2	0.7	4.6	**15.9**	3.2	—	-0.6	0.4	12.9	**13.3**	4.0	1.1	8.2	**8.1**	**51.2**
41-50	**21.2**	11.7	1.5	8.0	**21.5**	2.6	—	3.4	0.7	14.8	**10.2**	2.1	1.4	6.7	**1.8**	**45.6**
51-60	**36.3**	22.2	5.8	8.3	**19.6**	3.9	—	1.1	4.9	9.7	**3.2**	0.8	1.2	1.2	**1.9**	**39.4**
61-70	**49.6**	26.3	10.3	13.0	**14.9**	1.9	—	1.3	2.5	9.2	**1.5**	-0.6	0.3	1.8	**2.0**	**32.0**
All ages ≤ 50	**17.1**	9.1	1.7	6.3	**22.1**	2.6	—	2.4	3.9	13.2	**10.5**	2.8	1.4	6.3	**3.5**	**46.8**
All ages ≤ 70	**39.1**	21.1	8.3	9.7	**30.6**	4.4	—	1.9	4.1	20.2	**2.2**	0.3	0.6	1.3	**2.1**	**26.0**

*The contribution of a specific cause of death to differences in overall mortality by workforce participation was determined by expressing the rate difference of that specific cause (ARD_S) as a percentage of the rate difference of total mortality (ARD_T), (that is, ARD_S/ARD_T*100).

† CVD, cardiovascular disease

‡ International Classification of Diseases (ICD) codes; cardiovascular disease (codes 390 to 459 (ICD 8th and 9th version) or I00 to I99 (ICD-10th version)), coronary heart disease (410 to 414 (ICD 8th and 9th version) or I20 to I25 (ICD-10th version)), stroke (430 to 438 (ICD 8th and 9th version) or I60 to I69 (ICD-10th version)), cancer (140 to 239 (ICD 8th and 9th version) or C00 to D48 (ICD-10th version)), lung cancer (162 (ICD 8th and 9th version) or C34 (ICD-10th version)), prostate cancer (185 (ICD 8th and 9th version) or C61 (ICD-10th version)), breast cancer (174 (ICD 8th and 9th version) or C50 (ICD-10th version)), colorectal cancer (153 to 154 (ICD 8th and 9th version) or C18 to C22 (ICD-10th version)), psychiatric diseases (290 to 319 (ICD 8th and 9th version) or F00 to F99 (ICD-10th version)), external causes (800 to 999 (ICD 8th and 9th version) or S00 to T98 and V01 to Y98 (ICD-10th version)). suicide (E 950 to E959 (ICD 8th and 9th version) or X60 to X84 (ICD-10th version)) and traffic accidents (E807 to E846 (ICD 8th and 9th version) or V01 to V99 (ICD-10th version)). Those aged 20 years or less were classified according to the workforce participation of the household.
